# The effect of sunlight exposure on interleukin-6 levels in depressive and non-depressive subjects

**DOI:** 10.1186/1471-244X-13-75

**Published:** 2013-03-05

**Authors:** Rosa Levandovski, Bianca Pfaffenseller, Alicia Carissimi, Clarissa S Gama, Maria Paz Loayza Hidalgo

**Affiliations:** 1 Laboratório de Cronobiologia do Hospital de Clínicas de Porto Alegre (HCPA), da Universidade Federal do Rio Grande do Sul (UFRGS), Porto Alegre, Brazil; 2 INCT for Translational Medicine, Laboratory of Molecular Psychiatry, HCPA/UFRGS, Porto Alegre, Brazil; 3 Departamento de Psiquiatria e Medicina Legal da Faculdade de Medicina, da Universidade Federal do Rio Grande do Sul, Porto Alegre, Brazil; 4 Programa de Pós-Graduação em Ciências Médicas: Psiquiatria, UFRGS, Porto Alegre, Brazil; 5 Programa de Pós-Graduação em Medicina: Ciências Médicas, UFRGS, Porto Alegre, Brazil

**Keywords:** Interleukin, Light, Depressive disorder, Munich Chronotype Questionnaire (MCTQ)

## Abstract

**Background:**

The objective of this epidemiological study was to evaluate the effect of length of sunlight exposure on interleukin 6 (IL-6) levels in depressive and non-depressive subjects.

**Methods:**

This was a cross-sectional study with 154 subjects (54 males, mean age: 43.5 ± 12.8 years) who were living in a rural area in south Brazil. Chronobiological and light parameters were assessed using the Munich Chronotype Questionnaire. Sleep quality was evaluated using the Pittsburgh Sleep Quality Index. Depressive symptoms were assessed with the Beck Depression Inventory. Plasma levels of inflammatory cytokines (IL-2, IL-4, IL-6, IL-10, tumor necrosis factor-α, and interferon) were collected during the daytime and measured.

**Results:**

IL-6 levels showed a positive correlation with light exposure (r = 0.257; p < 0.001) and a negative correlation with the mid-sleep phase on work-free days (r = -0.177; p = 0.028). Multiple linear regression analysis showed that only the length of light exposure was an independent factor for predicting IL-6 levels (ß = 0.26; p = 0.002). In non-depressed subjects, exposure to a different intensity of light did not affect IL-6 levels (t = -1.6; p = 0.1). However, when the two depressive groups with low and high light exposure were compared, the low light exposure group had lower levels of IL-6 compared with the high light exposure group (t = -2.19 and p = 0.0037).

**Conclusions:**

The amount of time that participants are exposed to sunlight is directly related to their IL-6 levels. Additionally, depressed subjects differ in their IL-6 levels if they are exposed to light for differing amounts of time.

## Background

The pro-inflammatory cytokine interleukin-6 (IL-6) is involved in the regulation of several physiological processes, particularly in the immune response, as well as in sleep regulation [1], metabolism [2,3], and mood disorders [4,5].

IL-6 is produced by a variety of cells, including mononuclear phagocytes, T cells, fibroblasts, astrocytes, and microglia cells [6]. Although the light/dark circadian rhythm has been shown to regulate IL-6, only a few studies have been performed in humans. Diurnal variation of IL-6 has been reported in healthy subjects [7,8] and also in some clinical conditions, such as rheumatoid arthritis, myocardial infarction, and sleep deprivation [9-12]. Administration of a synthetic nuclear receptor involved in the circadian clockwork, the Rev-Erb ligand, modulates the production and release of IL-6 in human macrophages [13].

Diurnal variation of IL-6 to the light/dark rhythm is modulated by melatonin, which has been implicated in the production of IL-6 [14]. The suprachiasmatic nucleus (SCN) may be responsible for this modulation. The SCN sends information regarding the dark–light cycle to the paraventricular nucleus of the hypothalamus, to the lateral column of the spinal cord, and to the pineal gland to modulate melatonin release [15]. The melatonin rhythm plays the role of an endogenous synchronizer, which peaks at night and is able to entrain rhythms, such as temperature, hormone release, and the sleep-wake cycle via receptors in the SCN [15,16]. We have previously shown that, in depressed patients, excretion of the major melatonin urinary metabolite, 6-sulfatoxymelatonin (aMT6s), increases after antidepressant use. The higher the increment in aMT6s excretion, the better the antidepressant response of the drug, indicating an enhancement in pineal production of melatonin overnight [17]. Additionally, this effect may be related to changes in phase or poor sleep quality found in depressed patients [18] or subjects with greater levels of depressive symptoms [19].

Catecholamines also play an important role in the release of melatonin. Norepinephrine, which is an important neurotransmitter involved in mood disorders and pathophysiology, is involved in the regulation of the enzymatic activity that converts serotonin in N-acetylserotonin, the immediate precursor of melatonin [20]. Furthermore, catecholamines may exert a direct effect on the immunoinflammatory system, given that IL-6 release is stimulated by noradrenaline, whereas IL-6 inhibits the secretion of dopamine and serotonin [21,22].

Proinflammatory cytokines are thought to be involved in the pathogenesis of depression. However, previous studies have reached divergent conclusions about the relationship between depression and the levels of IL-6. Increased IL-6 levels have been observed during depression [4,23-28]. However, psychological symptoms, such as a negative mood, tension, and fatigue, are significantly associated with a greater nocturnal decrease of IL-6 levels [29]. IL-6, melatonin, cortisol, and depressive symptoms are not significantly altered by light therapy [4,30]; however, natural light exposure improves depressive symptoms [30].

Based on this evidence, we hypothesized that IL-6 levels are directly correlated with sunlight exposure and not just with artificial light exposure. Therefore, this epidemiological study evaluated the effects of the duration of sunlight exposure on IL-6 levels in depressive and non-depressive subjects.

## Methods

### Study population

**Figure 1:**
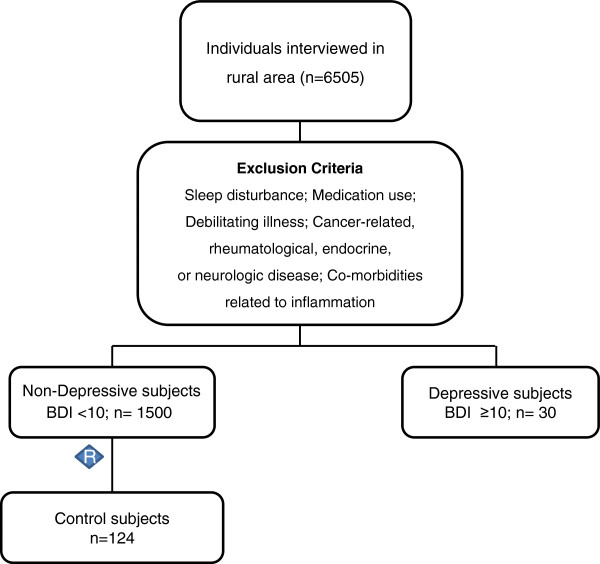
Flowchart of the inclusion procedure.

This was a cross-sectional study of an epidemiological sample (i.e., homogeneous in terms of culture and socio-economic level), living in a rural area in the south of Brazil. Notably, this area was unaffected by the nocturnal light pollution found in urban areas. The sample comprised 154 subjects with a mean age of 43.5 ± 12.8 years, ranging from 18 to 65 years of age. All subjects with a Beck Depression Inventory (BDI) ≥10 were selected from a larger sample of 6505 interviewed subjects. A total of 124 healthy subjects were randomly selected for the current study from 1500 subjects without a personal history of disease (Figure 1).

Exclusion criteria included sleep disturbance, cancer-related, rheumatological, endocrine, or neurological disease, co-morbidities related to inflammation, as well as the use of anti-inflammatory, antihypertensive, and psychopharmacological drugs, and cortisol. The study was conducted according to international ethical standards protocols [31] (ethics approval number: 08–087 GPPG/HCPA), and all participants provided informed consent.

### Instruments

Chronobiological and light parameters were assessed using the Munich Chronotype Questionnaire (MCTQ). Sleep quality was evaluated using the Pittsburgh Sleep Quality Index (PSQI), and depressive symptoms were evaluated with the BDI.

Research assistants blinded to the objective of the study and previously trained to avoid bias in measurements collected our data. Interviews were held during the day in the participant’s house. To guarantee blinding, the questionnaires contained several questions that were not related to depressive symptoms. Questionnaires were answered individually, following the instructions for each question.

### The MCTQ

We used the validated Brazilian-Portuguese version of the MCTQ (http://www.bioinfo.mpg.de/wepcronotipo/) to assess the mid-sleep phase on work-free days (MSFsc), average sleep duration, and average light exposure. The MCTQ assesses actual sleep times separately for work and work-free days. The MSFsc was calculated as the point of the mid-sleep phase on a work-free day. Sleep duration was calculated by averaging the estimates of sleep duration for both work and work-free days, over the course of 1 week [32]. The questionnaires provided information regarding sleep times, such as bed and rise times, plus the clock time of becoming fully awake, as well as sleep latency and inertia. In addition, the instrument has questions related to the total time of exposition to natural light, during work, and work-free days.

### Outdoor sunlight exposure measurements

Outdoor light exposure was assessed using the validated version of the MCTQ. The questionnaire includes questions related to the total time of exposure to sunlight, during work, and work-free days. The time of light exposure is self-reported using a standard question: “How long per day do you spend on average outside (really outside) exposed to day light? On work days: ___ h ___min and on free days: ___ h ___min.”

The average phase of sunlight exposure was defined by the average amount of time an individual was exposed to outdoor light, and was calculated by averaging the information for work and work-free days throughout the week using the equation: number of work days per week × number of hours reported on work days + number of work-free days per week × number of hours reported on work-free days [33].

### The PSQI

The PSQI [34] was used to measure sleep quality in this study. The PSQI version used was validated for Brazilian Portuguese language [35]. This index is composed of 15 multiple-choice items that inquire about the frequency of sleep disturbances, the subjective sleep quality, as well as four items that inquire about typical bedtime, time of awakening, sleep latency, and sleep duration. The PSQI generates seven subscores that correspond to the domains of the global score. Each component score ranges from 0 (indicating no difficulty) to 3 (indicating severe difficulty). The component scores are summed to obtain a global score (range of 0–21). A score > 5 is suggestive of significant sleep complaints.

### The BDI

We chose the validated Brazilian Portuguese version of the BDI for the screening of depressive symptoms [36,37]. This is a self-report scale that assesses cognitive, affective, and somatic symptoms of depression. The inventory consists of 21 items, with each question assigned to a score ranging from 0 (no symptoms) to 3 (severe symptoms), with the total BDI score ranging from 0 to 63. A total score of 0–9 indicates no or minimal depressive symptoms, 10–17 indicates mild depressive symptoms, 18–29 indicates moderate depressive symptoms, and 30–63 indicates severe depressive symptoms [38].

### Cytokine measurements

Inflammatory cytokine levels (IL-2, IL-4, IL-6, IL-10, tumor necrosis factor-alpha [TNF-α], and interferon) were assayed in plasma, which was collected during the day. Five milliliters of blood was collected by venipuncture into an EDTA vacuum tube. Immediately after withdrawal, the blood was centrifuged, and the plasma was aliquoted and stored at -80°C until later assay. Plasma cytokine levels were determined by flow cytometry using the BD™ Cytometric Bead Array (CBA) Human Th1/Th2/Th17 Cytokine Kit (BD Biosciences, San Diego, CA). The CBA kit allows for the discrimination of the following cytokines: IL-2, IL-4, IL-6, IL-10, interferon-gamma, TNF-α, and interferon. Sample processing and data analysis were performed according to the manufacturer's instructions. Briefly, plasma samples were incubated with cytokine capture beads for 1.5 h, washed, and then incubated for 1.5 h with PE-conjugated detection antibodies. Both incubations were performed at room temperature and protected from the light. The samples were then washed, and sample data were acquired using a FACSCalibur flow cytometer (BD Biosciences). The results were generated in both graphical and tabular formats using the BD CBA Analysis Software FCAP Array™ (BD Biosciences).

### Statistical analysis

Logarithmic transformations were completed for the following variables because they showed asymmetric distributions: IL-2, IL-4, IL-6, IL-10, TNF-α, interferon, and BDI. Spearman’s test was used to analyze the correlation among ILs and sleep parameters. To control for potential confounding effects, variables were analyzed by logistic regression analysis.

Average light exposure was categorized into two groups based on the 75^th^ percentile: low and high light exposure. Additionally, BDI scores were categorized into two groups: less than 10 (non-depressive) and equal to or greater than 10 (depressive). A two-tailed Student’s *t*-test for independent samples was used to analyze the mean difference between groups. For all analyses, the statistical significance was set at p <0.05. Data were analyzed using SPSS version 18.0 (SPSS, Chicago, IL).

## Results

With regard to symptoms of depression, approximately 19.5% of the sample population scored ≥10 on the BDI scale. The maximum score was 34 points, corresponding to mild to moderate depressive symptoms. Demographics, sleep characteristics, and anthropometric data of all subjects included in this study (n = 154) are shown in Table 1. The depressive group and non-depressive group were not different in terms of age (44.0 ± 12.28 vs 43.4 ± 12.95 years; t = -0.27; p = 0.78) and body mass index (25.2 ± 3.9 vs 25.6 ± 4.7; t = 0.42; p = 0.68). Moreover, mean IL-6 levels were not significantly different between women and men (1.3 ± 1.1 vs 1.1 ± 1.0 pg/ml; t = 1.14; p = 0.26). Therefore, these variables were not included in multivariate analyses because they were not potential confounding factors.

**Table 1:** Sample characteristics (n = 154)

***Variable***	***Mean ± Sd/n(%)***
Age	43.49 ± 12.8
Sex (Male*)*	54 (34.8%)
BDI score	5.9 ± 5.9
Body mass index	25.6 ± 4.6
Formal education (years)	7.7 ± 3.6
MSFsc	3.34 ± 1.7
Average Light Exposure	8.70 ± 2.7
PSQI score	5.12 ± 4.1
Average Sleep Duration	7.84 ± 1.3
*Beck Depression Inventory*	
BDI <10	124 (80.5%)
BDI >10	30 (19.5%)

Values are means and standard deviation; BDI, Beck Depression Inventory; MSFsc, mid-sleep phase on work-free days sleep corrected; PSQI, Pittsburgh Sleep Quality Index.

**Figure 2:**
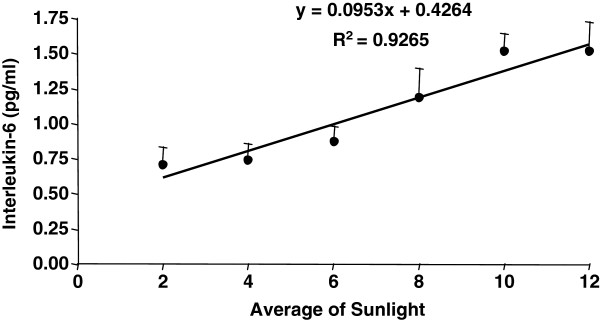
**The logarithmic values of IL-6 levels were plotted against the average sunlight exposure. **There was a positive correlation between IL-6 levels and average light exposure. IL-6 levels are shown in pg/ml. IL = interleukin.

Upon examination of the entire sample, a positive correlation was found between IL-6 levels and the average amount of sunlight exposure time (Spearman’s r = 0.26; p = 0.001; Figure 2), and a negative correlation was found between IL-6 levels and MSFsc (Spearman’s r = -0.177; p = 0.028; Table 2). However, there were no correlations among the average sleep duration, quality of sleep based on PSQI scores, and cytokine parameters (Table 2).

**Table 2:** Correlations between cytokines, chronobiological, and sleep variables

	**MSFsc**	**Average light exposure**	**Average sleep duration**	***PSQI score***
	**Spearman**	**Spearman**	**Spearman**	**Spearman**
	**( *p * value)**	**( *p * value)**	**( *p * value)**	**( *p * value)**
IL-2*	0.063 (0.44)	-0.026 (0.75)	-0.040 (0.63)	-0.063 (0.44)
IL-4*	-0.030 (0.71)	0.0659 (0.43)	-0.039 (0.63)	-0.015 (0.86)
IL-6*	**-0.177 (0.03)**	**0.256 (0.001)**	-0.019 (0.82)	0.050 (0.54)
IL-10*	0.034 (0.68)	0.022 (0.79)	0.057 (0.48)	-0.060 (0.46)
TNF-α*	-0.071 (0.38)	0.076 (0.35)	-0.038 (0.64)	0.066 (0.42)
Interferon*	0.062 (0.44)	-0.011 (0.90)	**-0.146 (0.07)**	-0.022 (0.78)

MSFsc = mid-sleep phase on work-free days sleep corrected; PSQI = Pittsburgh Sleep Quality Index; IL = Interleukin. * Variables log transformed.

To control for potential multicollinearity effects among variables, we analyzed the data with multivariate regression analysis, with covariate factors, including chronobiological, sleep, and light parameters, predicting the outcome variable, IL-6. The only dependent variable that was retained in the model was the average amount of sunlight exposure time. Therefore, MSFsc was considered a confounding variable (β = -0.05; p = 0.57). The average amount of sunlight exposure accounted for variance in IL-6 levels (β = 0.26; p = 0.002) (F = 6.304; R^2^ adjusted = 0.065; p = 0.002).

**Figure 3:**
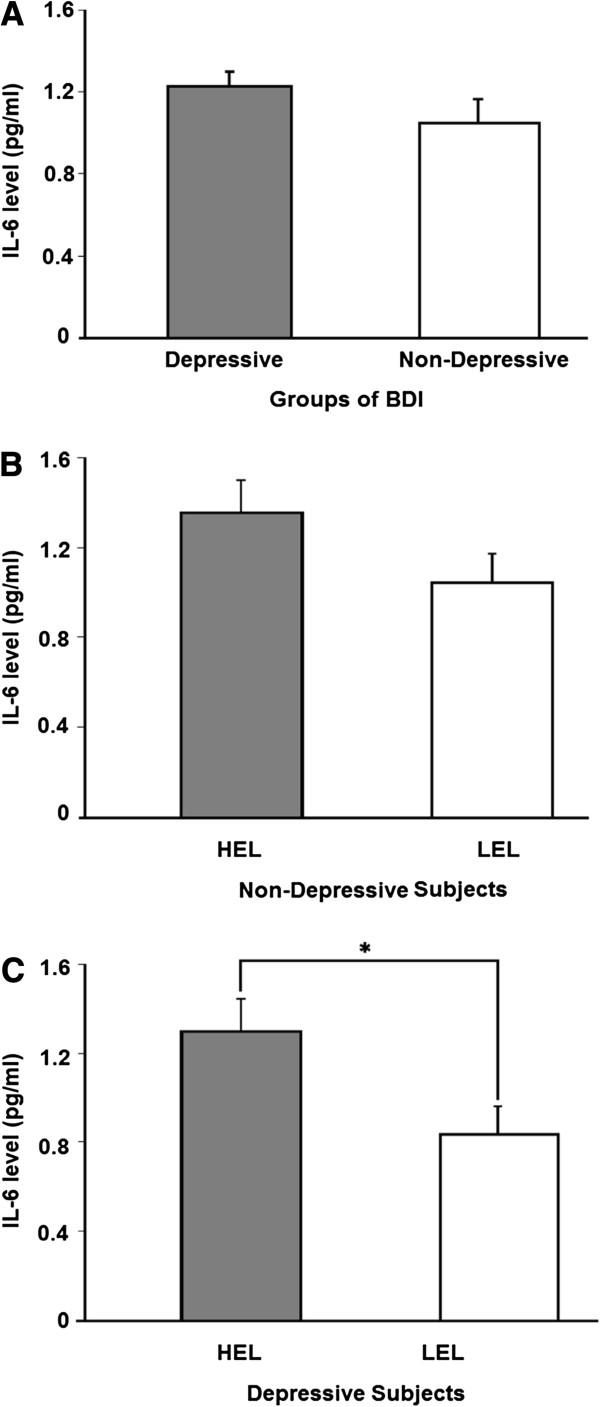
**A- Levels of IL-6 in relation to BDI scores (including the depressive and non-depressive groups). B- **Comparison of IL-6 levels between two groups: high exposure sunlight group (HEL) and low exposure sunlight group (LEL) in non-depressive subjects. **C**- Comparison of IL-6 levels between the HEL and LEL groups in depressive subjects. The Student’s *t*-test for independent samples was used to analyze the mean differences between groups. IL-6 = interleukin; IL-6 levels are shown in pg/ml. *p < 0.05.

IL-6 levels were not different among the depressive and non-depressive groups (Figure 3A). In the non-depressive group, comparison of IL-6 levels in subjects with high and low light exposure yielded no group differences (t = -1.6; p = 0.1; Figure 3B). However, when the low and high light exposure groups were compared in subjects with depressive symptoms, the low light exposure group showed lower levels of IL-6 compared with the high light exposure group (t = -2.19 and p = 0.0037; Figure 3C). This finding indicates that, when subjects are depressed, the length of sunlight exposure is related to IL-6 levels.

## Discussion

In this epidemiological study, our findings showed that the amount of sunlight exposure time was directly related to plasma IL-6 levels. Moreover, depressive subjects with high amounts of light exposure had significantly greater mean IL-6 levels compared with depressive subjects with low light exposure. These results are in accordance with the notion that exposure to sunlight is one of the most powerful factors that affects multiple physiological processes in humans, including the immune system.

Exposure to natural light has been the focus of human studies to understand the physiological effects of various 24-h patterns on the light–dark cycle, in contrast to effects of artificial light [39]. Our results demonstrated that depressed subjects differed in their IL-6 levels based on differing amounts of sunlight exposure. Behavior promoting natural light exposure is a simple and low-cost therapeutic practice to alleviate depression [30]. Therefore, the mechanism that links the therapeutic effect of light in depression may be the immune system, specifically by IL-6.

Our study reassessed the hypothesis that the link between depressive symptoms and immune responses is related to functioning of the SCN [40] and immune-pineal-axis, which are highly sensitive to light [20,41]. Unfortunately, we did not assess melatonin levels in our study. Therefore, this is an important hypothesis to be tested in a clinical study. Recently, inflammatory signals have been linked to the pineal gland, which produces melatonin. The immune-pineal-axis is one of the regulatory mechanisms responsible for the regulation of the inflammatory system by melatonin production [20].

Humans are likely to differ in their response to light depending on impairment of their brain, and therefore, depressed patients may be more prone to being reactive to light. This relationship can be explained by findings from previous reports, such as the demonstration that IL-6 has a protective effect at lower concentrations in brain tissue, but a neurotoxic effect at high concentrations [42]. However, even though IL-6 is a proinflammatory cytokine, it is not always beneficial and it is involved in acute inflammatory disease. Patients with acute episodes and those with sepsis have intermediate levels of interleukins, showing a systemic toxicity with mood episodes, compared with healthy subjects [43,44]. In a clinical study, patients with depressive episodes demonstrated an increase in IL-6 levels, whereas patients with maniac episodes showed an increase in IL-2, IL-4, and IL-6 levels [45].

*In vitro,* IL-6 promotes neuronal survival [46], exerts neuroprotective properties in cells against cytotoxic insults [47], and influences synaptic plasticity, as suggested by the modulation of synaptic potentiation in the hippocampus [48], with subsequent promotion of memory formation [49]. We hypothesize that the regulatory process of IL-6, promoted by the SCN, does not occur in the same manner in depressed patients compared with controls.

We did not assess the intensity or the quality of light using an objective instrument. Nevertheless, our sample was composed of rural individuals who worked outside. Therefore, it is likely that they are exposed to sunlight for longer amounts of time and are exposed to a different quality of light compared with European populations. In Brazil (30°S; 51W), the temperature is high and the photoperiod is long. Given that light exposure is mediated by numerous factors, evaluation of the quantity of sunlight exposure, as well as the intensity and quality of light in field work, should be performed in future studies.

The divergent findings related to IL-6 levels in mood disorders in the literature may be a consequence of differences in the methods and samples used. In our study, we chose only one time point for blood collection to assess cytokine levels throughout the day, and this may have limited our conclusions because of IL-6 rhythmicity in peripheral blood, which may be altered by day length. Nevertheless, our results demonstrated that, based on a positive linear correlation, IL-6 levels increased proportionally to longer periods of sunlight exposure. In addition, our sample was composed of mild and moderately depressed subjects who had never undergone treatment. Therefore, there was no effect of medication on IL-6 in this sample. The potential confounding effects of quality and phase of sleep were also controlled in our study.

## Conclusions

The amount of time that participants are exposed to sunlight is directly related to their IL-6 levels. Additionally, depressed subjects differ in their IL-6 levels based on the duration of sunlight exposure. To the best of our knowledge, this is the first epidemiological study examining the crosstalk between circadian variations in sunlight and immune system parameters in depressive and non-depressive subjects. These findings underscore the hypothesis that the beneficial effect of sunlight exposure on depressive disorders is attributable to protection by ILs. We hypothesize that differences in IL-6 levels in depressive subjects are a result of impairment of the central nervous system, more specifically, the SCN.

## Abbreviations

IL-6: Interleukin 6; MCTQ: Munich chronotype questionnaire; BDI: Beck depression inventory; SCN: Suprachiasmatic nucleus; (aMT6s): 6-sulfatoxymelatonin; PSQI: Pittsburgh sleep quality index; MSFsc: Mid-sleep phase on work-free days sleep corrected; CBA: Cytometric bead array.

## Competing interests

The authors declare that they have no competing interests.

## Authors’ contributions

RL and MPLH designed the study, wrote the protocol, and performed literature searches and analyses. RL, AC, and MPLH wrote the first draft of the manuscript. BP and CSG performed cytokine measurements, quality control, and the final revision of the manuscript. All authors contributed to and have approved the final manuscript. RL (Rosa Levandovski); MPLH (Maria Paz Loayza Hidalgo); AC (Alicia Carissimi); BP (Bianca Pfaffenseller); CSG (Clarissa S Gama).

## Pre-publication history

The pre-publication history for this paper can be accessed here:

http://www.biomedcentral.com/1471-244X/13/75/prepub
